# An amylin analogue attenuates alcohol-related behaviours in various animal models of alcohol use disorder

**DOI:** 10.1038/s41386-019-0323-x

**Published:** 2019-01-23

**Authors:** Aimilia Lydia Kalafateli, Daniel Vallöf, Giancarlo Colombo, Irene Lorrai, Paola Maccioni, Elisabet Jerlhag

**Affiliations:** 1 Department of Pharmacology, Institute of Neuroscience and Physiology, The Sahlgrenska Academy at the University of Gothenburg, Gothenburg, Sweden; 2 Neuroscience Institute, Section of Cagliari, National Research Council of Italy, Monserrato, CA Italy

**Keywords:** Gene expression analysis, Reward, Behavioural methods

## Abstract

Recent findings have identified salmon calcitonin (sCT), an amylin receptor agonist and analogue of endogenous amylin, as a potential regulator of alcohol-induced activation of the mesolimbic dopamine system and alcohol consumption. Providing that the role of amylin signalling in alcohol-related behaviours remains unknown, the present experiments investigate the effect of sCT on these behaviours and the mechanisms involved. We showed that repeated sCT administration decreased alcohol and food intake in outbred rats. Moreover, single administration of the potent amylin receptor antagonist, AC187, increased short-term alcohol intake in outbred alcohol-consuming rats, but did not affect food intake. Acute administration of sCT prevented relapse-like drinking in the “alcohol deprivation effect” model in outbred alcohol-experienced rats. Additionally, acute sCT administration reduced operant oral alcohol self-administration (under the fixed ratio 4 schedule of reinforcement) in selectively bred Sardinian alcohol-preferring rats, while it did not alter operant self-administration (under the progressive ratio schedule of reinforcement) of a highly palatable chocolate-flavoured beverage in outbred rats. Lastly, we identified differential amylin receptor expression in high compared to low alcohol-consuming rats, as reflected by decreased calcitonin receptor and increased receptor activity modifying protein 1 expression in the nucleus accumbens (NAc) of high consumers. Collectively, our data suggest that amylin signalling, especially in the NAc, may contribute to reduction of various alcohol-related behaviours.

## Introduction

The 37 amino-acid hormone, amylin, is cosecreted with insulin in the pancreatic β-cells and subsequently released in the blood [1]. Physiologically, it inhibits insulin secretion, gastric emptying, and glucagon secretion [2]. In addition, amylin and its analogue and receptor agonist salmon calcitonin (sCT) reduce food intake by signalling satiation [3–7]. On a similar note, central [8] or intravenous [3] administration of the potent amylin receptor antagonist AC187 [2] increases food intake, confirming the role of the endogenous amylin pathway in food intake control. These anorexigenic effects seem to be centrally mediated via amylin receptors in various brain areas [9–11], including the area postrema, nucleus of the solitary tract, and dorsal raphe among others [12]. It was recently identified that sCT also reduces food intake and energy balance by acting on amylin receptors in areas associated with reinforcement of alcohol and other substances, namely the nucleus accumbens (NAc), ventral tegmental area (VTA), and laterodorsal tegmental area (LDTg) [13–15]. More specifically, it does so by activating amylin receptors located on ventral tegmental dopaminergic neurons [16, 17].

In attempt to better understand the neurochemical substrates of alcohol-related behaviours and to possibly develop novel medications for alcohol disorders, studies have tried to identify novel modulators of alcohol reward [18, 19]. On that note, peptides controlling food intake and energy balance have an important role as reinforcement regulators of alcohol and other addictive substances [20–28]. Despite the little evidence supporting amylinergic regulation of artificial rewards, sCT has been shown to attenuate the rewarding properties of alcohol in rodents, and more specifically to decrease alcohol-induced locomotor activity, accumbal dopamine release, and conditioned place preference in mice, as well as alcohol intake in rats [29]. Providing that the role of amylin signalling in alcohol-related behaviours still remains unidentified, we aimed to investigate the effect of sCT on additional alcohol-related behaviours in rats that reflect various aspects of alcohol use disorder (AUD) in humans. Firstly, we explored the effects of repeated administration of sCT on alcohol and food intake in outbred rats. Secondly, we investigated the ability of single administration of AC187 to affect alcohol and food intake in outbred rats to further confirm a role of circulating amylin in alcohol intake in rats. Additionally, we investigated whether acute sCT administration affects relapse-like drinking in outbred rats using the “alcohol deprivation effect”(ADE) model and the effect of acute sCT injection on operant oral alcohol self-administration (under the fixed ratio (FR) 4 (FR4) schedule of reinforcement) in selectively bred Sardinian alcohol-preferring (sP) rats (for review, see [30]). Moreover, we tested the ability of acute administration of sCT to affect operant self-administration (under the progressive ratio (PR) schedule of reinforcement) of a highly palatable chocolate-flavoured beverage in outbred rats. Lastly, to identify neurocircuits involved in amylinergic alcohol-induced reward regulation, we investigated the expression patterns of the main components of the amylin receptor, namely the calcitonin receptor (*CALCR*) and receptor activity modifying proteins 1 and 3 (*RAMP1* and *RAMP3*), in reward-related brain areas of high and low alcohol-consuming rats.

## Materials and methods

(*For detailed protocols see* Supplementary Information)

### Animals

For the alcohol intake and gene expression experiments (including ADE) conducted in the Swedish laboratory, adult post-pubertal age-matched male outbred Rcc Han Wistar rats (Envigo; Horst, The Netherlands) were used since they display a voluntary high and stable alcohol intake causing pharmacological relevant blood alcohol concentrations in the intermittent access model [31]. The rats were housed individually in high Macrolon III cages covered with filter tops (Tecniplast, Buguggiate, Italy) and maintained at 20 °C with 50% humidity.

In the Italian laboratory, male sP rats, from the ninety-sixth generation and 60-days-old at the start of the study, were used in the operant alcohol self-administration experiments. Moreover, male Wistar rats (Harlan Laboratories, San Pietro al Natisone, Italy), of ~60 days of age at the start of the study, were used in the operant chocolate self-administration experiments. Both sP and Wistar rats were group-housed per three in standard plastic cages (Tecniplast, Buguggiate, Italy) under a constant temperature of 22 °C and relative humidity of 60%. In both laboratories, rats were maintained on a 12 h reversed light/dark cycle (lights off at 8 and 7 am in the Swedish and Italian lab, respectively). Food and water were available *ad libitum* in the homecage, except for short periods during the initial phase of training in the operant self-administration paradigm as noted. The experiments were approved by the Swedish Ethical Committee on Animal Research in Gothenburg or the Ethical Committee of the University of Cagliari. All efforts were made to minimise animal suffering, and to reduce the number of animals used. Each experiment used an independent set of rats. All animals were allowed to acclimatise at least 1 week before the start of the experiments. Before the start of the study, rats were extensively habituated to handling and intraperitoneal injections.

### Drugs

sCT (Tocris Bioscience, Bristol, United Kingdom) was diluted in vehicle (0.9% sodium chloride solution), and administered intraperitoneally (IP) at the doses of 1 or 5 μg/kg always 30 min prior to alcohol presentation. AC187 (Tocris Bioscience, Bristol, United Kingdom) was diluted in vehicle (0.9% sodium chloride solution) and administered at a dose of 250 μg/kg (IP), 5 min prior to alcohol presentation, in order to compensate for the drug’s short half-life and probable short bioavailability [3, 7].

### Intermittent access 20% alcohol two-bottle-choice drinking paradigm in outbred rats

The rats were given free access to one bottle of 20% (v/v) alcohol (96%, VWR International AB, Stockholm, Sweden) and one bottle of water during three 24-h sessions per week (Mondays, Wednesdays, and Fridays), while on the other days of the week were provided with unlimited access to two water bottles (Tuesdays, Thursdays, and the weekend). During 12 baseline weeks, all bottles were weighed at 24 h after the fluids were placed to the rat cages. The body weight of each rat was measured daily prior to bottle presentation, to allow for calculating the grams of pure alcohol intake per kilogram of body weight (g/kg). The average baseline consumption of the rats was calculated and the treatment design was balanced for future experiments.

#### Alcohol intake following repeated administration of sCT

In the first drinking experiments, rats were administered once-daily single injection of sCT (5 μg/kg, IP) or vehicle solution (saline solution, IP) on three subsequent alcohol-drinking days (Monday, Wednesday, and Friday). In this experiment, the parameters measured at 1 and 24 h were alcohol intake, alcohol preference, water intake, total fluid intake, and food intake. Body weight was measured at 24-h time points and the body weight change was then calculated. In addition, the 24-h water intake of the untreated days was measured.

#### Alcohol intake following acute administration of AC187

A separate group of rats was subjected to a single injection of AC187 (250 μg/kg, IP) or equal volume of vehicle solution (saline solution, IP) on an alcohol-drinking day (Monday and Wednesday), in a balanced design. There was 1-day break between each administration and each animal served as its own control. Alcohol intake, alcohol preference, water intake, total fluid intake, and food intake were measured at 1, 4, and 24 h, with the additional time point of 4 h reflecting the drug’s short half-life [8]. Body weight was measured at 24-h time points and the body weight change was then calculated. In addition, the 24-h water intake of the untreated days was measured.

#### Acute administration of sCT on ADE

The ADE model has been established after evidence that, following forced abstinence in alcohol-experienced rats, voluntary alcohol consumption will increase compared to baseline drinking conditions [32]. In the present study, rats were subjected to the intermittent access 20% alcohol two-bottle-choice drinking paradigm (as described earlier) for 10 consecutive weeks. Thereafter, the rats were deprived of alcohol for 10 days, and alcohol was thereafter reintroduced. Thirty minutes before the reintroduction of alcohol, the rats were treated with either sCT (5 μg/kg, IP, *n* = 20) or vehicle (IP, *n* = 18) in a balanced design and the 24-h alcohol intake was then measured.

#### CALCR, RAMP1, and RAMP3 expression in low and high alcohol-consuming rats

Following 12 weeks of intermittent access to alcohol, rats were decapitated and the brains were removed and immediately placed on a cold glass plate. The NAc, VTA, amygdala, hippocampus, prefrontal cortex, and striatum were rapidly dissected, transferred into a plastic tube and stored in −80 °C until further analysis. Real-time qPCR was performed to identify the expression levels of the main components of the amylin receptor *CALCR*, *RAMP1*, and *RAMP3* (genes of interest, GOI). In this experiment the selected reference gene (RG) was *GAPDH*. The TaqMan® Gene Expression Essays used for screening in these experiments were as follows: Rn01427056_m1 (*RAMP1* rat), Rn00571815_m1 (*RAMP3* rat), Rn00587525_m1 (*CALCR* rat), and Rn01775763_g1 (*GAPDH* rat) (Thermo Fisher Scientific, Waltham, MA, USA).

### Operant alcohol self-administration and body weight change in sP rats

The self-administration experiments were performed in the Italian lab as previously described in Maccioni et al., 2015 (for detailed protocol information, see Supplementary information). This experiment allowed the assessment of the effect of sCT on the reinforcing properties of alcohol (beside its mere consumption). Briefly, the test session took place the day after termination of the 20-day maintenance phase. In the test session, response requirement (RR) on the alcohol and water lever was kept at FR4 and FR1, respectively. The test session lasted for 30 min. Vehicle or sCT, at the doses of 1 and 5 μg/kg, was administered IP to independent groups of rats (*n* = 12 per group). Measure variables were the number of lever-responses and amount of self-administered alcohol (g/kg). The day after the test session, rats were exposed to an additional self-administration session. Body weight was measured prior to the test session as well as 24 h later; body weight change was subsequently calculated.

### Operant chocolate self-administration and body weight change in outbred rats

The operant chocolate self-administration study was performed as previously described in detail [33] (for detailed protocol information, see Supplementary information). This experiment was conducted with the intent of adding experimental evidence to the possible anorectic profile of sCT by accounting for the motivational properties of palatable food.

Briefly, the test session took place the day after termination of the 20-session maintenance phase under the FR10 schedule of reinforcement. The test session lasted for 60 min and occurred under the PR schedule of reinforcement. Vehicle or sCT, at the doses of 1 and 5 μg/kg, was administered IP to independent groups of rats (*n* = 12 per group). Measure variables were the number of lever-responses and breakpoint for the chocolate-flavoured beverage. The day after the test, rats were exposed to one subsequent self-administration session under the FR10 schedule of reinforcement. Body weight was measured prior to the test session as well as 24 h after; body weight change was subsequently calculated.

### Statistical analysis

Comparisons of average baseline values for the design of future treatment groups in both the repeated treatment and ADE experiments were analysed with an unpaired *t*-test. The data from the repeated treatment using the intermittent alcohol access model were evaluated using a repeated two-way ANOVA followed by Bonferroni’s multiple comparisons test, to assess effect of treatment and time. The effect of acute AC187 treatment on rats in the intermittent alcohol access model was evaluated by a paired *t*-test. The analysis of baseline and ADE values within the treatment groups was assessed with a paired two-tailed *t*-test, whereas the baseline and ADE values between treatment groups were analysed with an unpaired two-tailed *t*-test. The effect of acute treatment on operant alcohol and chocolate self-administration, as well as on body weight change, was analysed using an ordinary one-way ANOVA followed by Tukey’s test for multiple comparisons. The overall effect of acute treatment and time on ADE was assessed with a two-way ANOVA followed by Bonferroni’s post hoc test. For the gene expression data, an unpaired *t*-test of the experimental ΔC_T_ values was performed. Data are presented as mean ± SEM. A probability value of *P* < 0.05 was considered as statistically significant.

## Results

### Repeated treatment of sCT reduces alcohol intake and increases body weight change in outbred rats

**Fig. 1:**
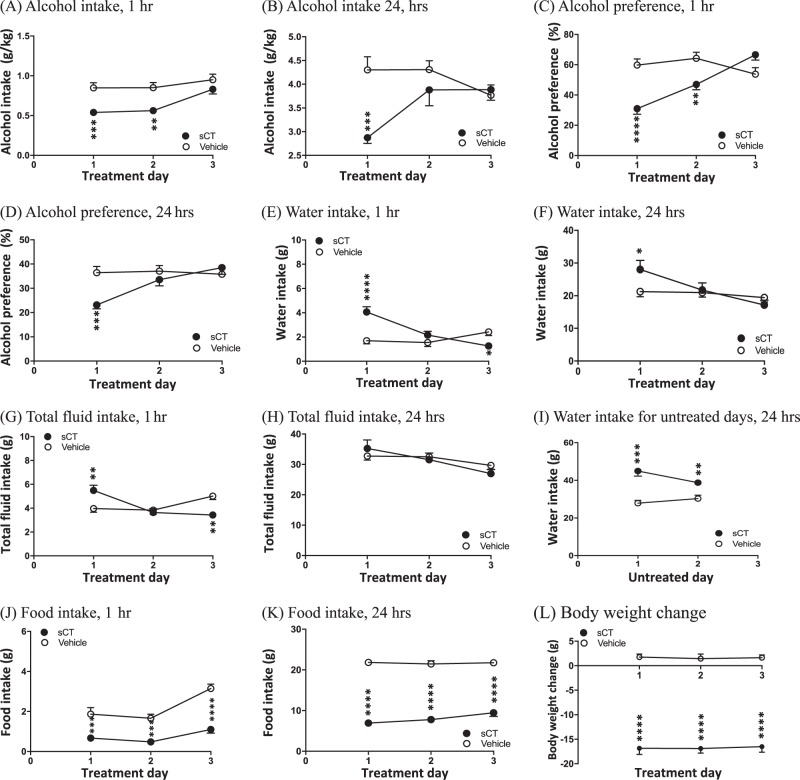
Repeated sCT administration attenuates voluntary alcohol intake, alcohol preference, and food intake and decreases body weight in outbred rats. **a** Repeated sCT treatment (5 μg/kg, IP) reduced alcohol intake in rats (*N* = 25) in the intermittent access 20% alcohol two-bottle-choice drinking paradigm at the time point of 1 h on the first and second treatment days compared to vehicle (*N* = 25). **b** Repeated sCT administration reduced alcohol intake at the time point of 24 h on the first treatment day. sCT reduced alcohol preference at the time point of **c** 1 h on treatment days 1 and 2 and at the time point of **d** 24 h on treatment day 1. sCT increased water intake at the time point of **e** 1 h on treatment day 1, but reduced water intake on treatment day 3 when compared to vehicle. At **f** 24 h, the sCT group showed increased water intake only on the first treatment day. Repeated sCT injections decreased total fluid intake at the time point of **g** 1 h on the first treatment day, but decreased water intake on the and last treatment day when compared to vehicle. sCT administration had no effect on water at the time point of **h** 24 h on any treatment day. sCT increased **i** 24-hour water intake during the untreated days when compared to vehicle. Repeated sCT injections (5 μg/kg, IP) decreased food intake in rats at the time point of **j** 1 h and **k** 24 h on all treatment days when compared to vehicle. **l** Repeated sCT administration increased the 24-hour values of percentage of body weight change. (Data are presented as mean ± SEM; *P < 0.05, **P < 0.001, ***P < 0.001, ****P < 0.0001; white circle indicates vehicle, black circle indicates sCT)

After 12 weeks of intermittent alcohol intake, the 24-h average baseline alcohol consumption of the rats that would receive either sCT or vehicle was similar (3.70 ± 0.20 g/kg, *N* = 25 for the vehicle group and 3.75 ± 0.21 g/kg, *N* = 25 for the sCT group, *P* = 0.7494). Repeated administration of sCT (5 μg/kg, IP) on 3 alcohol days revealed an overall effect of treatment (F(1, 48) = 15.28, *P* = 0.0003) and of time (F(2, 96) = 11.14, *P* < 0.0001) on alcohol intake, but there was no significant effect between treatment x time interaction. sCT decreased alcohol intake compared to vehicle on treatment day 1 (*P* < 0.0001) and 2 (*P* < 0.001), but not on day 3 (*P* > 0.05) as shown in Fig. 1a. The 24-h values analysis did not show an overall effect of treatment (F(1, 48) = 2.35, *P* = 0.1318), however, an effect of time (F(2, 96) = 14.24, *P* < 0.0001) and treatment x time interaction (F(2, 96) = 15.68, *P* < 0.0001) on alcohol intake was noted. Further analysis showed that sCT treatment decreased 24-h alcohol intake compared to vehicle, but only on treatment day 1 (*P* < 0.0001), as shown in Fig. 1b.

Alcohol preference was overall affected by sCT-repeated treatment (F(1, 48) = 8.68, *P* = 0.005) for the 1-h time point. Time (F(2, 96) = 9.75, *P* = 0.0001) and treatment x time interaction (F(2, 96) = 19.5, *P* < 0.0001) had an overall effect on alcohol preference. sCT administration attenuated alcohol preference on treatment days 1 (*P* < 0.0001) and 2 (*P* < 0.01), but not on treatment day 3 (*P* > 0.05) compared to controls, as shown in Fig. 1c. The treatment did not have an effect (F(1, 48) = 2.35, *P* = 0.1318) on alcohol preference after analysis of the 24-h scores as shown in Fig. 1d. However, there was an overall effect of time (F(2, 96) = 14.24, *P* < 0.0001) and treatment x time interaction (F(2, 96) = 15.68, *P* < 0.0001). sCT administration decreased 24-h preference for alcohol on treatment day 1 (*P* < 0.001) compared to vehicle controls, but not on treatment days 2 and 3 (*P* > 0.05).

Analysis of 1-h water intake (Fig. 1e) revealed an overall effect of treatment (F(1, 48) = 4.11, *P* = 0.0481), time (F(2, 96) = 8.60, *P* = 0.0004), and treatment x time interaction (F(2, 96) = 18.79, *P* < 0001). Rats administered sCT consumed significantly more water compared to controls on treatment day 1 (*P* < 0.0001). However, data from treatment day 3 revealed that water values were higher in vehicle-treated animals (*P* < 0.05), while no significant differences were noted on day 2 (*P* > 0.05). Twenty four-h water intake analysis (Fig. 1f) revealed no overall effect of treatment (F(1, 48) = 0.55, *P* = 0.4633); however, an effect of time (F(2,96) = 20.73, *P* < 0.0001) and time x treatment interaction (F(2, 96) = 10.88, *P* < 0.0001). Water intake was significantly increased in the sCT group when compared to vehicle on day 1 (*P* < 0.05), but no differences were noted on days 2 and 3.

Analysis of the total fluid intake scores at the 1-h time point (Fig. 1g) revealed no effect of treatment (F(1, 48) = 0.07, *P* = 0.7921). However, an effect of time (F(2, 96) = 5.71, *P* = 0.0045) and time x treatment interaction (F(2,96) = 13.88, *P* < 0.0001) was noted. On treatment day 1, the sCT group showed significantly higher consumption of fluids (*P* < 0.01) compared to the vehicle group, while on day 2 there was no significant difference noted (*P* > 0.05). Finally, on day 3, the sCT group showed lower total fluid consumption (*P* < 0.01). There was an overall effect of time (F(2, 96) = 15.74, *P* < 0.0001) and time x treatment interaction (F(2, 96) = 3.34, *P* = 0.0395), but not of treatment (F(1, 48) = 0.03, *P* = 0.8594) on the total fluid consumption of the rats measured at the 24-h time point (Fig. 1h). There were no statistical differences between the groups at any given treatment day (*P* > 0.05 for all days).

An overall effect of treatment (F(1, 48) = 29.96, *P* < 0.0001) as well as time x treatment interaction (F(2, 96) = 8.08, *P* = 0.0066), but not of time (F(2, 96) = 1.51, *P* = 0.2245), was noted in the 24-h values of the total water intake during the untreated days (Fig. 1i). The analysis of water consumption on the days when the rats did not receive any pharmacological treatment showed increased water consumption for the sCT group compared to vehicle on both untreated no-alcohol days 1 and 2 (*P* < 0.0001 and *P* < 0.01, respectively).

### Repeated treatment of sCT reduces food intake and increases body weight change in outbred rats

There was an overall main effect of treatment (F(1, 48) = 51, *P* < 0001), time (F(2, 96) = 19.68, *P* < 0.0001), and time x treatment interaction (F(2, 96) = 3.89, *P* = 0.0237) on food intake rat at the 1-h time point (Fig. 1j). sCT significantly decreased 1-hour food intake compared to vehicle on all treatment days 1–3 (*P* < 0.001, *P* < 0.001, and *P* < 0.0001, respectively). The 24-hour food intake scores revealed an effect of treatment (F(1, 48) = 463.70, *P* < 0.0001), but no effect of time (F(2, 96) = 2.02, *P* = 0.1387) or time x treatment interaction (F(2, 96) = 2.00, *P* = 0.1405). Treatment with sCT significantly reduced food intake at the 24-hour time point (Fig. 1k) and the effect was noted on all treatment days (*P* < 0.0001 for all days).

Lastly, Fig. 1l shows the analysis of the grams of body weight change, which revealed an overall main effect of treatment (F(1, 48) = 511.6, *P* < 0.0001), but no effect of time (F(2, 96) = 0.05, *P* = 0.9539) or time x treatment interaction (F(2, 96) = 0.03, *P* = 0.9714). Further analysis revealed that, in the sCT group, the grams of body weight change at 24 h was significantly higher compared to vehicle on all treatment days (*P* < 0.0001 for all days).

### Acute AC187 administration increases short-term alcohol intake, but does not affect food intake in outbred rats

**Fig. 2:**
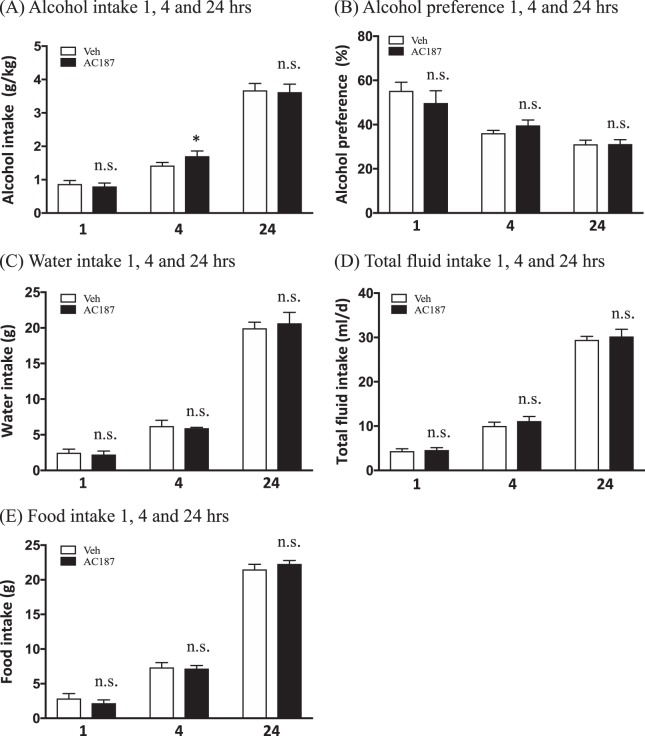
AC187 increases short-term alcohol intake, but does not affect food intake in outbred rats. Single administration of AC187 (250 μg/kg) did not affect **a** alcohol intake in outbred rats (*N* = 9) compared to vehicle (Veh) (*N* = 9) at the time point of 1 h, but significantly increased alcohol consumption at the time point of 4 h. There was no difference in long-term alcohol intake 24 h after AC187 administration. AC187 did not have an effect on **b** alcohol preference scores at any time point of 1 h, 4 h, or 24 h. No effect was noted on **c** water intake at any measured time of 1 h, 4 h, or 24 h, similarly to **d** total fluid intake scores for 1, 4, and 24 h, respectively. AC187 did not affect **e** food intake at the time point of 1 h, 4 h, or 24 h. (Data are presented as mean ± SEM; *P < 0.05, n.s.: *P* > 0.05)

Following 12 weeks of intermittent alcohol intake, the 24-h average baseline alcohol consumption of the rats was 4.62 ± 0.04 g/kg (*N* = 9). Acute treatment with a single injection of AC187 (250 μg/kg) did not affect alcohol intake at the time point of 1 h (*P* = 0.3684, *n* = 9, Fig. 2a), but significantly increased alcohol consumption compared to vehicle at 4 h (*P* = 0.0392, Fig. 2a). AC187 did not affect long-term alcohol intake at the time point of 24 h as shown in Fig. 2a (*P* = 0.7356). Additionally, AC187 administration did not affect alcohol preference scores at any time point measured of 1 h (*P* = 0.4208), 4 h (*P* = 0.2016), or 24 h (*P* = 0.9480) as shown in Fig. 2b.

The water intake scores were not affected by AC187 administration at the 1-h (*P* = 0.6958), 4-h (*P* = 0.7427), or 24-h (*P* = 0.5514) time points (Fig. 2c). There was no difference in total fluid intake between rats treated with AC187 compared to vehicle, 1 h (*P* = 0.7054), 4 h (*P* = 0.3084), or 24 h (*P* = 0.5490) after AC187 administration (Fig. 2d).

As shown in Fig. 2e, single injection of AC187 did not affect food intake scores at any measured time point of 1 h (*P* = 0.1019), 4 h (*P* = 0.7358), or 24 h (*P* = 0.1940). Lastly, the 24-h measured body weight change was unaffected by AC187 compared to rats that received vehicle injections (*P* = 0.9386; data not shown).

### Acute sCT administration prevents ADE in outbred rats

The baseline 24-h alcohol intake values of the rats later treated with vehicle (3.53 ± 0.22 g/kg; *N* = 18) were not statistically different (*P* = 0.8248) when compared to those of the later sCT-treated group (3.60 ± 0.21 g/kg; *n* = 20).

**Fig. 3:**
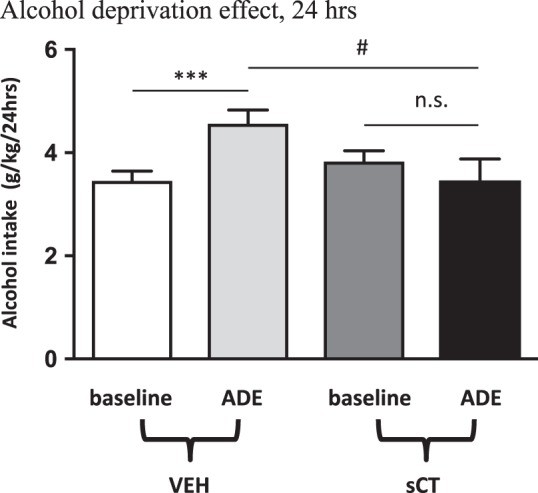
sCT prevents ADE in outbred rats. A single injection of sCT (5 μg/kg) prevents the alcohol deprivation effect (ADE) in rats (*N* = 18) abstained from alcohol for 10 days. There was an increase in alcohol intake post abstinence in the vehicle (Veh) group, but not in the sCT group when compared to baseline values. (Data are presented as mean ± SEM; ***P < 0.001 for baseline vs sCT within the Veh group, ^#^*P* < 0.05 for ADE vs ADE between the Veh-sCT groups, n.s.: *P* > 0.05)

Analysis of the 24-h values of alcohol intake in the ADE session (Fig. 3) showed no main effect of treatment (F(1, 36) = 2.75, *P* = 0.1054) or time (F(1,36) = 2.20, *P* = 0.1459) but an overall effect of treatment x time interaction (F(1, 36) = 8.19, *P* = 0.0070). Further analysis revealed development of ADE (i.e. significantly higher alcohol intake) in vehicle receiving (*P* = 0.0007), but not in sCT-receiving rats (*P* = 0.4125). The baseline 24-h alcohol intake values before the abstinence period for the ADE experiments, did not statistically differ between the two treatment groups (*P* = 0.3204). The alcohol intake values after abstinence were significantly higher in the vehicle-treated when compared to the sCT-treated group (*P* = 0.0149).

### Acute sCT administration decreases operant alcohol self-administration and increases body weight change in sP rats

**Fig. 4:**
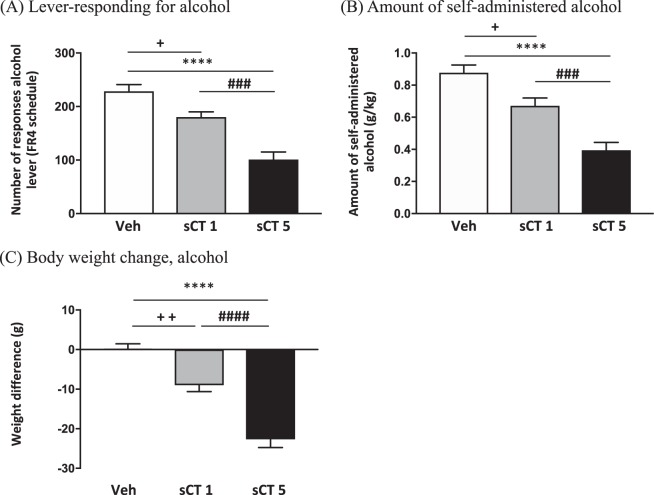
sCT administration decreases lever-responding for alcohol and amount of self-administered alcohol in sP rats. **a** Acute sCT administration in the dose of 1 μg/kg (sCT1) (*N* = 12) and 5 μg/kg (sCT5) (*N* = 12) decreased the number of lever-responses for alcohol in a dose-response manner when compared to vehicle (Veh) (*N* = 12) in Sardinian alcohol-preferring (sP) rats exposed to the FR4 schedule of reinforcement. **b** Acute administration of sCT in the same doses reduced the amount of self-administered alcohol in a dose-response manner. **c** In the same experiment, body weight change (in grams) was increased by acute administration of sCT in two doses in a dose response-like effect. (Data are presented as mean ± SEM;^+^*P* < 0.05,^+^^+^*P* < 0.01 for Veh vs sCT1, ****P < 0.0001 for Veh vs sCT5 and ^###^*P* < 0.001, ^####^*P* < 0.0001 for sCT1 vs sCT5)

Acute treatment with two doses of sCT (1 and 5 μg/kg, IP) showed an overall effect on the number of lever responses for alcohol in sP rats (*N* = 12 per group; (F(2, 33) = 28.17, *P* < 0.0001; Fig. 4a). Further analysis revealed that sCT at the dose of 1 μg/kg significantly decreased the number of lever responses compared to vehicle (*P* < 0.05). A dose of 5 μg/kg significantly attenuated the number of lever-responses (*P* < 0.0001) when compared to vehicle. The effect of sCT was dose dependent, as the high dose of sCT (5 μg/kg) significantly reduced the number of lever responses when compared to the low (1 μg/kg; *P* < 0.0005).

An overall main effect of treatment (F(2, 33) = 24.77, *P* < 0.0001) was noted in the amount of self-administered alcohol (Fig. 4b). Rats receiving sCT at the dose of 1 μg/kg, self-administered a significantly lower amount of alcohol when compared to controls (*P* < 0.05). Similarly, rats receiving the high dose of sCT (5 μg/kg) self-administered significantly less alcohol than vehicle-treated rats (*P* < 0.0001). In addition, there was a significant difference in the amount of self-administered alcohol between the groups receiving the low and the high dose of sCT (*P* < 0.001). Lever responding for water was negligible (averaging <2 in all rat groups) and not altered by treatment with sCT (*P* = 0.6300; data not shown). No difference on the number of lever responses for alcohol and the amount of self-administered alcohol was recorded among the rat groups treated with 0, 1, and 5 μg/kg sCT in the self-administration session conducted the day after the test session (*P* = 0.26; data not shown).

Acute administration of two doses of sCT (1 and 5 μg/kg) had an overall effect (F(2, 33) = 45.79, *P* < 0.0001) on body weight difference (expressed in grams) of the sP rats, as shown in Fig. 4c. Acute administration of a low dose of sCT (1 μg/kg) significantly increased body weight change compared to vehicle (*P* < 0.01). Body weight change was significantly greater after administration of a high dose of sCT (5 μg/kg) when compared to vehicle (*P* < 0.0001). Additionally, the group receiving a high dose of sCT (5 μg/kg) showed a significant increase in body weight change compared to the group receiving a low (1 μg/kg; *P* < 0.0001).

### Acute sCT administration does not affect operant self-administration of a chocolate-flavoured beverage, but increases body weight change in outbred rats

Acute treatment with two doses of sCT (1 and 5 μg/kg, IP) did not have any effect (F(2, 33) = 0.36, *P* = 0.6977) on the number of lever responses for the chocolate-flavoured beverage in Wistar rats (*N* = 12 per group; Supplementary figure 1A). Accordingly, there was no effect of treatment (F(2, 33) = 0.38, *P* = 0.6842) on the breakpoint for the chocolate-flavoured beverage, as shown in Supplementary figure 1B. No difference on the number of lever responses for the chocolate-flavoured beverage and the amount of self-administered chocolate-flavoured beverage was recorded among the rat groups treated with 0, 1, and 5 μg/kg sCT in the self-administration session conducted the day after the test session under the FR10 schedule of reinforcement (*P* = 0.69; data not shown).

There was an overall main effect of treatment (F(2, 33) = 11.66, *P* < 0.0001) on body weight change (expressed in grams) (Supplementary figure 1C). Compared to vehicle, the low dose of sCT (1 μg/kg) did not affect body weight change (*P* > 0.05). Body weight change was significantly increased in rats treated with the high dose of sCT (5 μg/kg) compared to vehicle (*P* < 0.001). Lastly, there was a significant difference in the percentage of body weight change between the rats treated with a low (1 μg/kg) and a high (5 μg/kg) sCT dose (*P* < 0.01).

### Effect of long-term alcohol exposure on the main amylin receptor components, *CALCR*, *RAMP1*, and *RAMP3* in rats

**Fig. 5:**
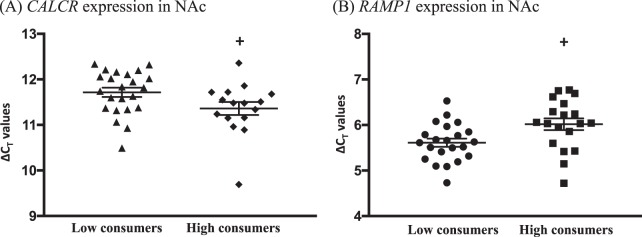
The expression of the *CALCR* gene in NAc is lower in high alcohol-consuming rats, whereas the expression of *RAMP1* is higher in the same brain area. **a** The expression of *CALCR* in NAc is decreased in high (*N* = 19) compared to low alcohol-consuming (*N* = 25) rats. Conversely, **b** the expression of *RAMP1* in the same area is higher in high compared to low alcohol-consuming rats. (Data are presented as mean ± SEM of the ΔC_T_ values; ^**+**^*P* < 0.05)

Alcohol consumption decreased the expression of the *CALCR* in the NAc, in high compared to low alcohol-consuming rats (cut off 3.5 g/kg/24 h; low consumers *n* = 24, high consumers *n* = 19; *P* = 0.0434, Fig. 5a). In the same area, higher expression of the *RAMP1* was noted in high compared to low alcohol-consuming rats (low consumers *N* = 25, high consumers *N* = 19; *P* = 0.0115, Fig. 5b), whereas no differences were noted in the expression of *RAMP3* in the same area in either group (low consumers *N* = 25, high consumers *N* = 19; *P* = 0.2154, data not shown). There were no differences noted in the expression of the *CALCR* (Supplementary Table 1), *RAMP1* (Supplementary Table 2), or *RAMP3* (Supplementary Table 3) in the dorsal striatum, VTA, and amygdala. Although qPCR was performed in the hippocampus and prefrontal cortex for the detection of the *CALCR* levels, due to lack of sufficient yielding C_T_ values for these areas, further analysis is not included in this manuscript.

## Discussion

AUD is a complex neuropsychiatric disorder with great impact on individual health and society, however, the pharmacotherapies currently available show different response between individuals [19]. Therefore, we here present data indicating the potential involvement of amylinergic receptor activation by sCT in alcohol-related behaviours, which reflect AUD components, in rats.

In the present study, we found that repeated sCT administration for a period of 3 days decreased alcohol intake, as well as alcohol preference, both short term (1 h data) and long term (24 h data) in rats. This is in line with our previous work, where acute administration of the same dose of sCT reduces alcohol intake at the 1-h time point in low alcohol-consuming outbred rats, with a more pronounced reduction in high alcohol-consuming outbred rats that is present at the 24-h time point [29]. Seemingly, the present data indicate that the effect of sCT on alcohol intake shows a tolerance pattern, which is in line with previous data showing that repeated sCT infusions caused a tolerance pattern in regards to food intake and body weight [34]. Interestingly, the selectivity of tolerance towards alcohol intake possibly indicates that food intake and alcohol reward are modulated by different systems and they show differential sensitivity to sCT. Nevertheless, our results could open the way for the use of other synthetic amylin agonists in the treatment of AUD, which do not demonstrate this tolerant pattern.

The present data also provide the first indication that amylinergic receptor activation after a single injection of sCT prevents the occurence of ADE in alcohol-abstained rats. ADE in rodents is an important reflection of relapse caused by alcohol craving in patients with AUD [32]. Providing that current available treatments for AUD prevent ADE in rats [35] as well as relapse caused by craving in humans [36], the present data show promising clinical relevance for development of new pharmacotherapies.

We demonstrate for the first time that acute treatment with sCT dose dependently decreases the number of lever responses for alcohol and amount of self-administered alcohol in selectively bred alcohol-preferring sP rats, suggesting that at least in this rat line the reinforcing properties of alcohol are likely under amylinergic regulation. These data are of importance, as operant self-administration tasks in rodents reflect the motivational aspect to consume a reinforcer [37], modeling a different facet of AUD. The dose-response relationship reported in the operant alcohol self-administration procedure is further supported by our previous study where we found that this low dose of sCT (1 μg/kg) reduced, but did not antagonise, alcohol-induced locomotor stimulation [29].

The amylinergic system has been implicated in natural reward regulation, as shown by amylin receptor regulation of sexual behaviour in rats by inhibiting dopamine transmission [38], and by activation of dopamine D2 receptors in area postrema neurons in regards to amylin-signalled satiation [39]. Regarding substance reward, recent findings support that sCT attenuates the well-documented effects of alcohol on the mesolimbic dopamine system, like locomotor activity, accumbal dopamine release, and condition placed preference in mice [29]. Accordingly, treatment with sCT has been reported to block amphetamine-induced locomotor stimulation in rats [40]. Moreover, a recent study showed a trend of decreased amylin levels after cocaine intravenous injection in cocaine-experienced users [41].

Albeit the limited data linking amylinergic receptor activation and alcohol-related behaviours, indirect data have showed lower levels of the calcitonin gene-related peptide (a calcitonin family member) in the hippocampus and frontal cortex of alcohol-preferring P rats compared to NP rats [42], providing a tentative molecular background of amylinergic regulation of alcohol drinking. Interestingly, peripherally administered sCT does not alter food intake in rats with VTA-knocked-down calcitonin receptors, but does so in controls [17], proposing that sCT can effectively pass the blood–brain barrier to reach the areas of the mesolimbic dopamine system. Our present findings showing that two components of the amylin receptor, namely *CALCR* and *RAMP1*, are expressed differently in the NAc of low and high alcohol-consuming rats, propose a differential receptor signaling, which depends on chronic alcohol consumption. NAc has been characterized as an intense amylin [14], as well as sCT’s site of action [14, 43], where all the components of functional amylin receptors are expressed [12, 44], primarily comprising of the CTR-A-RAMP1 complex [45, 46]. A possible hypothesis could support that chronic alcohol consumption affects amylin receptor activity by altering its components’ expression in the NAc. This could potentially influence the dopaminergic activity in the area, leading to differential behavioral responses to alcohol’s rewarding properties. On the other hand, there were no differences in amylin receptor expression of other investigated areas, indicating that amyling singlling in NAc plays a key role in alcohol intake. Despite the small difference in the expression patterns of the amylin receptor in the NAc in this study, these changes reflect the involvement of this area in amylin-modulated alcohol reward, in accordance to our previous data showing that sCT blocks alcohol-induced accumbal dopamine release [29]. It should also be considered that amylin receptors in other areas may contribute to high alcohol consumption. For example, Indiana alcohol-preferring (P) and high alcohol-drinking (HAD) rats have fewer calcitonin gene-related peptide receptor-binding sites in the central amygdaloid nucleus and caudate putamen compared to non-preferring (NP) and low alcohol-drinking (LAD) rats [47]. Notably, more studies investigating the protein levels of the components of the amylin receptor will shed more light on the post-transcriptional status of the genes investigated here and they are warranted for the future.

Moreover, we showed that the potent antagonist, AC187, increased short term (4 h), but not long-term (24 h), alcohol intake. Despite the lack of evidence regarding whether AC187 crosses the blood–brain barrier, AC187 is a potent competitor for rat amylin-binding sites in brain areas like the nucleus accumbens, and inhibits metabolic responses of administered amylin in vivo [48]. Based on the above, we suggest that peripheral AC187 crosses the blood–brain barrier to reach areas of the mesolimbic dopamine system, causing increase of alcohol intake in rats. Interestingly, the time frame of the noted effect here is in line with studies remarking a peak significant increase of food intake 4 h after bolus infusion of AC187 into the third ventricle [8], supporting a role of endogenous circulating amylin in addiction processes. It should be considered here, that neurotransmitters like the calcitonin gene-related peptide (CGRP) [49] are ligands for amylin receptors and especially amylin receptors 1 and 2 [50]. Therefore, administration of AC187 could potentially antagonize CGRP’s function in the brain and influence the obtained results. Nevertheless, providing the involvement of amylin receptor activation in alcohol regulation and the noted increase in alcohol intake after AC187 administration, we suggest that this possibility unlikely affects alcohol-mediated behaviours.

The alteration in amylin receptor expression in the NAc in high-consuming rats could suggest that the remarked effects are not reward related, but influenced by other parameters. A tentative explanation could be that sCT influences alcohol consumption either metabolically or by causing an altered stress response. In previous experiments we showed that sCT administration does not alter alcohol concentration levels or corticosterone in the blood in mice, thus we can exclude the implication of a differential regulation of alcohol consumption [29]. Another possible factor influencing our data would be nausea or taste aversion caused by sCT administration. It has however been documented that peripheral amylin [5] or sCT [7] does not cause adverse effects like nausea or malaise. Potential caloric regulation of alcohol after sCT administration could also be a factor influencing our findings. Nevertheless, sCT seems to lack any effect on a highly caloric food like peanut butter in mice experiments [29] as well as on a chocolate-flavoured beverage (present study), therefore leading us to the hypothesis that the remarked effects on alcohol consumption are not calorically regulated. In the present study, sCT was administered peripherally, potentially implying the involvement of peripheral receptors in the obtained results. This seems highly unlikely, as we have peviously presented that sCT attenuates alcohol-induced reward, which is mediated via the mesolimbic dopamine system [29]. Nevertheless, additional studies with local administration of sCT into reward-related areas are of substantial interest.

We also noted that sCT has an effect on water intake both hourly and daily and that could be explained by compensation of the reduced alcohol intake, since the 24-h total fluid consumption is not affected. This is in line with our previous studies where a single injection of sCT increased water intake in a high alcohol-consuming group of outbred rats in the same paradigm [29]. Moreover, this drinking pattern is commonly observed in alcohol intake studies with ghrelin receptor antagonists and GLP-1 receptor agonists [51, 52].

The present data demonstrate a robust and persistent effect of sCT on hourly and daily values of food intake, as well as on increase of body weight change in both sP and outbred rats. This is in line with our previous studies, where sCT decreases 24-h values of body weight on high alcohol-consuming rats [29]. On the same note, more studies demonstrate the effect of amylin receptor activation on food intake [53, 54] and body weight [8]. Moroever, peripheral sCT decreases 24-h body weight in outbred [55] and diet-induced obese rats [56]. Additionally, pramlintide reduces body weight in overweight/obese and diabetic (type 2) patients with a pronnounced effect on obese patients [57]. This is corrobarated by our data from the operant self-administration experiments showing robust dose-response decrease of body weight. On a similar note, antagonism of amylin receptors in the brain increases food intake and body adiposity in rats, as a result of blocking of central amylin signalling systems [8]. Importantly, we argue that the short-term effects on body weight seen here, most likely do not reflect metabolic changes, but rather reflect differences in food intake. In the present study, we demonstrated that AC187 did not affect food intake and body weight of the rats. In accordance are data showing that chronic IP infusion of the antagonist affected food intake only in obese Zucker rats, but not in the respective lean controls, leaving also the body weight unaffected [58]. Furthermore, acute IP administration of AC187 alone was not able to alter food intake in food-restricted rats [7].

In the present study, we demonstrated that acute sCT administration had no effect on the motivational properties of a highly palatable chocolate-flavoured beverage in outbred rats in either dose administered. This is in accordance with our previous data showing that neither dose of sCT (1 or 5 μg/kg) affected peanut butter consumption as a palatable food in satiated mice [29]. Our results contradict to studies showing decrease of the number of lever responses for a milk-based reinforcer [59] and reduction of palatable food intake [60] in mice. However, different experimental setups were used, where the first study included amylin injections in fasted mice for 30 min [59], and the second was conducted in leptin-resistant mice [60].

Collectively, our results show for the first time that positive regulation of amylinergic receptors in rats attenuates several alcohol-related behaviours. The present findings suggest that central amylinergic pathways, especially in the NAc, are involved in the regulation of alcohol reinforcement, as well as in the development of different alcohol-related behaviours in rats. Providing that synthetic amylin agents as well as sCT products are already available for the treatment of diabetes [61], osteoporosis [62], and Paget’s disease [63], these or similar compounds render promising pharmacotherapiers for AUD.

## Funding and disclosure

EJ has received financial support from the Novo Nordisk Foundation. This does not alter the authors’ adherence to any of the journal’s policies on sharing data and materials. The remaining authors declare no conflict of interest. The study is supported by grants from the Swedish Research Council (2015-03219), Swedish Society for Medical Research, The Swedish brain foundation, LUA/ALF (grant no. 148251) from the Sahlgrenska University Hospital, Torsten Söderberg, Alcohol research council of the Swedish alcohol retailing monopoly and the foundations of Adlerbertska, Fredrik and Ingrid Thuring, Tore Nilsson, Längmanska, Wilhelm and Martina Lundgren, Knut and Alice Wallenberg, Magnus Bergvall, Anérs, Jeansons, Åke Wiberg, Novo Nordisk and the Swedish Society of Medicin.

## Supplementary information

Supplemental Tables 1, 2 & 3

Supplementary Material

Supplementary information

Supplementary information
