# Supplementary material for: An amylin analogue attenuates alcohol-related behaviours in various animal models of alcohol use disorder
Source: Neuropsychopharmacology. 2019 Jan 23;44(6):1093–102. doi: 10.1038/s41386-019-0323-x (PMC6461824; doi:10.1038/s41386-019-0323-x)
Supplement: Supplementary file 1 — Supplemental Tables 1, 2 & 3 [file 41386_2019_323_MOESM1_ESM.docx]

**TABLES**

**Table 1. *CALCR* expression in reward-related areas of high and low alcohol-consuming rats (cut off 3.5 g/kg/24hrs)**

| **Brain region** | **Low alcohol- consuming rats** | **High alcohol- consuming rats** | **t** | | **df** | | ***P*-value^1^** |
| --- | --- | --- | --- | --- | --- | --- | --- |
| **Dorsal striatum** | 15.25 ± 0.286, n=24 | 15.32 ± 0.223, N=18 | 0.1933 | | 40 | | 0.8477 |
| **Ventral tegmental area** | 1.17 ± 0.136, n=25 | 1.48 ± 0.186, N=21 | 1.3430 | | 44 | | 0.1863 |
| **Amygdala** | 13.56 ± 0.145, n=26 | 13.8 ± 0.159, N=19 | 1.0630 | 43 | | 0.2938 | |

Data presented as mean ΔC_T_ values ± SEM. ^1^unpaired t-test.

**Table 2. *RAMP1* expression in reward-related areas of high and low alcohol-consuming rats (cut off 3.5 g/kg/24hrs)**

| **Brain region** | **Low alcohol- consuming rats** | **High alcohol- consuming rats** | **t** | **df** | ***P*-value^1^** |
| --- | --- | --- | --- | --- | --- |
| **Dorsal striatum** | 4.96 ± 0.0836, n=26 | 5.16 ± 0.107, N=22 | 1.5060 | 46 | 0.1390 |
| **Ventral tegmental area** | 1.06 ± 0.0765, n=25 | 1.16 ± 0.116, N=21 | 0.7098 | 44 | 0.4816 |
| **Prefrontal cortex** | 7.83 ± 0.119, n=28 | 7.98 ± 0.075, N=20 | 0.9481 | 46 | 0.3480 |
| **Amygdala** | 7.19 ± 0.0744, n=26 | 7.25 ± 0.105, N=20 | 0.5132 | 44 | 0.6104 |
| **Hippocampus** | 9.05 ± 0.0673, n=24 | 9.17 ± 0.105, N=20 | 0.9403 | 42 | 0.3524 |

Data presented as mean ΔC_T_ values ± SEM. ^1^unpaired t-test.

**Table 3. *RAMP3* expression in reward-related areas of high and low alcohol-consuming rats (cut off 3.5 g/kg/24hrs)**

| **Brain region** | **Low alcohol- consuming rats** | **High alcohol- consuming rats** | **t** | **df** | ***P*-value^1^** |
| --- | --- | --- | --- | --- | --- |
| **Dorsal striatum** | 11.37 ± 0.142, n=26 | 11.79 ± 0.200, N=22 | 1.7530 | 46 | 0.0863 |
| **Ventral tegmental area** | 1.05 ± 0.12, n=23 | 1.46 ± 0.187, N=21 | 1.9110 | 42 | 0.0628 |
| **Prefrontal cortex** | 10.11 ± 0.211, n=28 | 10.07 ± 0.085, N=20 | 0.1411 | 46 | 0.8884 |
| **Amygdala** | 11.98 ± 0.118, n=27 | 11.98 ± 0.118, N=27 | 0.0855 | 44 | 0.9322 |
| **Hippocampus** | 12.4 ± 0.142, n=24 | 12.52 ± 0.227, N=20 | 0.4419 | 42 | 0.6608 |

Data presented as mean ΔC_T_ values ± SEM. ^1^unpaired t-test.
