# Supplementary material for: An amylin analogue attenuates alcohol-related behaviours in various animal models of alcohol use disorder
Source: Neuropsychopharmacology. 2019 Jan 23;44(6):1093–102. doi: 10.1038/s41386-019-0323-x (PMC6461824; doi:10.1038/s41386-019-0323-x)

Supplementary figure 1

(A) Lever-responding for chocolate

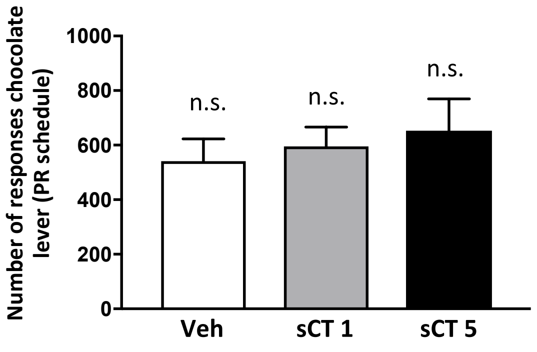

(B) Breakpoint for chocolate

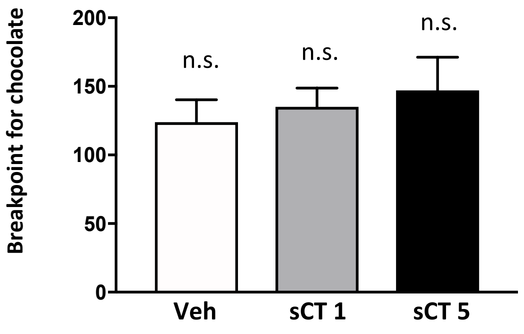

(C) Body weight change, chocolate

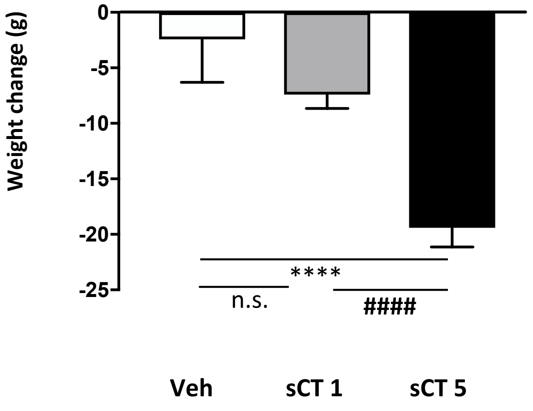

Supplement: Supplementary file 2 — Supplementary Material [file 41386_2019_323_MOESM2_ESM.pdf]
