# Supplementary material for: An amylin analogue attenuates alcohol-related behaviours in various animal models of alcohol use disorder
Source: Neuropsychopharmacology. 2019 Jan 23;44(6):1093–102. doi: 10.1038/s41386-019-0323-x (PMC6461824; doi:10.1038/s41386-019-0323-x)
Supplement: Supplementary file 3 — Supplementary information [file 41386_2019_323_MOESM3_ESM.docx]

**Suppl. Fig. 1. sCT administration does not affect lever-responding and breakpoint for a chocolate-flavoured beverage in outbred rats**

Compared to vehicle (Veh) (N=12), acute sCT administration at the doses of 1 μg/kg (sCT1) (N=12) and 5 μg/kg (sCT5) (N=12) did not affect (A) the number of lever responses for a chocolate-flavoured beverage and (B) breakpoint for a chocolate-flavoured beverage in Wistar rats exposed to a PR schedule of reinforcement. (C) In the same experiment, the low dose of sCT (1 μg/kg) did not affect, whereas the high dose (5 μg/kg) increased, body weight change (expressed in grams) (Data are presented as mean ±SEM; ***P<0.001 for Veh vs sCT5, ##P<0.01 sCT1 vs sCT5, n.s.: P>0.05)
