# Supplementary material for: An amylin analogue attenuates alcohol-related behaviours in various animal models of alcohol use disorder
Source: Neuropsychopharmacology. 2019 Jan 23;44(6):1093–102. doi: 10.1038/s41386-019-0323-x (PMC6461824; doi:10.1038/s41386-019-0323-x)
Supplement: Supplementary file 4 — Supplementary information [file 41386_2019_323_MOESM4_ESM.doc]

**SUPPLEMENTARY MATERIALS AND METHODS**

**Drugs**

The sCT doses were selected based on data from our previous dose response studies that showed no effect *per se* on baseline locomotor activity and gross behaviour of mice and rats and that sCT exerts its pharmacological action on alcohol-induced reward within this time interval [1]. The 1 μg/kg dose was considered as a low dose,since it reduced alcohol-induced locomotor stimulation, as opposed to the higher dose of 5 μg/kg, which completely attenuated the locomotor stimulation induced by alcohol and decreased alcohol intake in rodents [1].The dose of 5 μg/kg has also been extensively used in the literature as an effective dose in reducing food intake in rodents (Braegger et al, 2014; Lutz et al, 2000; Mietlicki-Baase et al, 2013).

The AC187 dose was selected based on previous studies showing that AC187 displaces sCT from calcitonin receptors in a 38-fold higher potency [2] and that 100:1 ratio of AC187 to amylin doses is hypothesized to be sufficient to cause amylin receptor antagonism [3]. A small pilot test of a higher dose of 500 μg/kg (IP), showed a decrease in water intake (data not shown) and was therefore not used in further experiments.

**Operant alcohol self-administration and body weight change in sP rats**

The self-administration chambers were located in sound-attenuated cubicles, with fans for ventilation and background white noise. The front panel of each chamber was equipped with (a) two retractable response levers, (b) one dual-cup liquid receptacle positioned between the two levers, and (c) two stimulus lights (one green and one white) mounted above each lever. A white house light was centred at the top of the back wall of each chamber. For half of the rats, the right lever was associated with alcohol, and achievement of the response requirement (RR) (a) activated the alcohol pump, resulting in the delivery of 0.1 ml alcohol solution, and (b) switched on the white light for the 2-s period of alcohol delivery; for these rats, the left lever was associated with water, and achievement of RR (a) activated the water pump, resulting in the delivery of 0.1 ml water, and (b) switched on the green light for the 2-s period of water delivery. The study design was counterbalanced so that the opposite condition was applied to the other half of the rats (left lever: alcohol; right lever: water).

Rats were initially exposed to the home cage two-bottle “alcohol vs water” choice regimen with unlimited access for 24-hours/day over 10 consecutive days. The alcohol solution was presented at the concentration of 10% (v/v). This initial phase was (a) part of the conventional procedure of alcohol self-administration employed in the Italian laboratory with sP rats (e.g.: Maccioni et al., 2015) and (b) conducted to allow the rats to become accustomed to the taste of alcohol and start to experience its pharmacological effects in order to possibly shorten the subsequent auto-shaping phase once rats are introduced into the operant chambers. During this phase, daily alcohol intake averaged approximately 6.5 g/kg.

Immediately after the two-bottle choice regimen, rats were introduced into the operant chambers and trained to lever-respond for alcohol. Self-administration sessions lasted 30 min (with the sole exception of the first session that lasted 120 min) and were conducted 5 days per week (Monday to Friday) during the first 4 to 6 hours of the dark phase of the daily light/dark cycle. Rats were deprived of water exclusively during the 12 hours prior to the first session in the operant chamber. Rats were initially exposed to a fixed ratio (FR) 1 (FR1) schedule of reinforcement for 10% alcohol (v/v) for 4 consecutive daily sessions. FR was then increased to FR2 and FR4 over 4 consecutive sessions. In sessions 9 and 10, the alcohol solution was presented at a final concentration of 15% (v/v). Rats were then exposed to 4 consecutive sessions during which the water lever alone or the alcohol lever alone was available every other day; water and alcohol were available on FR1 and FR4, respectively. From then onwards, both levers were concomitantly available (maintenance phase) for a total of 20 sessions conducted with FR4 and FR1 on the alcohol and water lever, respectively. After the 20 self-administration sessions of the maintenance phase, rats were selected for inclusion in the pharmacological experiment; only rats displaying a stability in lever-responding for alcohol of less than 25% daily difference over the last 5 daily sessions were selected.

**Operant chocolate self-administration and body weight change in outbred rats**

The chocolate-flavoured beverage was prepared as a solution of Nesquik® (Nestlè, Milan, Italy) chocolate powder in tap water in a concentration of 5% w/v. The operant chambers were equipped with (a) one retractable response lever, (b) one green stimulus light above the lever, (c) the retractable spout ofa liquid sipper bottle (250-ml capacity) located outside thechamber, and (d) a white house light centred at the top of the back wall. The lever was to the right ofthe retractable spout for half of the rats and in an oppositeposition (left) for the other half of the rats. Illumination of the house light signalled the start of the self-administration session. Achievement of RR resulted in 5-s exposure of the sipper bottle spout and illumination of the green lightfor the time period of spout exposure. Training took place during 5 weekdays, during the dark phase of the light/dark cycle. During the training phase the rats learned to lever-respond for the chocolate-flavoured beverage under the FR schedule of reinforcement. Self-administration sessions lasted 60 minutes. FR was progressively increased from FR1 to FR10 over 10 sessions and then maintained stable for 20 additional sessions (maintenance phase).

The effect of sCT on the motivational properties of the chocolate-flavoured beverage was assessed in a single test session conducted the day after termination of the maintenance phase. This test session was performed under the progressive ratio (PR) schedule of reinforcement; specifically, all testsession conditions (session length; chocolate powder concentration; duration of reinforcer presentation) were identical to those of maintenance sessions,with the sole exception of RR: the latter was increased progressivelyover the session as follows: 10, 12, 15, 20, 25, 32, 40, 50, 62, 77, 95, 118,

145, 178, 219, etc. [4], up to achievement of breakpoint, defined as the lowest RR not completed by the rat; breakpoint is a validated measure of the motivational properties of the reinforcer (Markou et al., 1993).

**Statistical analysis**

Comparisons of average baseline values for the design of future treatment groups in both the repeated treatment and ADE experiments were analysed with an unpaired t-test.The analysis of baseline and ADE values within the treatment groups was assessed with a paired two-tailed t-test, whereas the baseline and ADE values between treatment groups were analysed with an unpaired two-tailed t-test.

***CALCR*, *RAMP1* and *RAMP3* expression in low and high alcohol-consuming rats**

All surfaces and instruments were cleaned with RNase decontamination solution (RNase*Zap***™** , Invitrogen**™**, ThermoFisher Scientific, Wilmington, DE, USA). For RNA tissue preparation, the brain samples were homogenized by adding a TissueLyser II (Qiagen, Hilden, Germany). The total RNA was extracted using the RNeasy Lipid Tissue Mini kit (Qiagen) and samples were loaded on the QIAcube^TM^ (Qiagen, Hilden, Germany) for automated RNA extraction, following the manufacturer’s protocols. The quality and concentration of RNA were assessed in 1μl of the final RNA solution, using a NanoDrop 1000 Spectrophotometer (ThermoFisher Scientific, Wilmington, DE, USA). RNA concentration was calculated equally for all samples at 1000 ng per sample. After dilution with Milli-Q^TM^ (Millipore Corporation, Billerica, MA, USA) water, the samples were loaded in duplicates in a 96 well plate (Sarstedt AG & Co, Nümbrecht, Germany) and were prepared for a 20 μl reverse transcription reaction into cDNA using the QuantiTect Reverse Transcription kit (Qiagen, Hilden, Germany) as per manufacturer’s instructions. The quantitative real-time PCR (qRT-PCR) analysis was performed in the facilities of TATAA Biocenter AB Gothenburg, Sweden. Briefly, the samples were corrected for gDNA contamination using the ValidPrime^TM^ (TATAA Biocenter AB) technology and gene expression analysis was performed using the qRT-PCR instrument IntelliQube^TM^ (Douglas Scientific, Alexandria, MN, USA). The corrected C_T_ values raw data were analysed using the comparative C_T_ method as previously described [5]. The low alcohol-consuming rats were set as the internal calibrator. In brief, the individual ΔC_T_ values were calculated as: C_Τ (_*_GOI_*) –C_T (_*_average of RG_*).

**REFERENCES**

Kalafateli AL, Vallof D, Jerlhag E. Activation of amylin receptors attenuates alcohol-mediated behaviours in rodents. Addict Biol. 2018.

Young AA, Gedulin B, Gaeta LSL, Prickett KS, Beaumont K, Larson E, et al. Selective amylin antagonist suppresses rise in plasma lactate after intravenous glucose in the rat: Evidence for a metabolic role of endogenous amylin. FEBS Letters. 1994;3:237-241.

Reidelberger RD, Haver AC, Arnelo U, Smith DD, Schaffert CS, Permert J. Amylin receptor blockade stimulates food intake in rats. Am J Physiol Regul Integr Comp Physiol. 2004;3:R568-574.

Maccioni P, Colombo G (2017). Operant Self-Administration of Chocolate in Rats: An Addiction-Like Behavior. In: Philippu A (ed). *In Vivo Neuropharmacology and Neurophysiology*. Springer New York: New York, NY, pp 107-139.

Livak KJ, Schmittgen TD. Analysis of relative gene expression data using real-time quantitative PCR and the 2(-Delta Delta C(T)) Method. Methods (San Diego, Calif). 2001;4:402-408.
